# Dental Education

**Published:** 1867-02

**Authors:** 


### Editorial.

### DENTAL EDUCATION.

The accompanying "Resolutions" and suggestions have been issued by authority of the American Dental Association. It may be justly regarded as the sentiment of any truly professional man—of any one who has the progress and welfare of his profession at heart.

We would direct special attention to the extension of time of pupilage, and the classification of studies recommended. Formerly, two years were regarded as sufficient length of time for the Dental student to complete his course of study. There are upon the records of many societies, resolutions to this effect. But within the past few years a change has taken place, so that now many societies have made declarations in favor of an extension of time, and most of them, to the term of three years. This is doubtless a move in the right direction; though some students may be able to pass over the present prescribed curriculum in two years of close study, yet there will be many who fail to accomplish what should be done in three years, or even four. Three years is now regarded as the shortest average time practicable.

A point of almost vital importance is contained in the words, "He must possess at least a good English education."

The Dental profession has suffered perhaps more from the deficient education of its members, and those seeking to become its members, than from any other cause; and yet young men who are sadly deficient, yes, almost wholly wanting in a literary education, are encouraged and assisted to enter the ranks of the profession, by those of whom we should expect better things, and who are certainly conscious that they are inflicting a grievous outrage upon their chosen profession. But the question is asked, may not a man who is not possessed of a good education, be a good Dentist? Such an one may become by practice a good manipulator, but that will be based upon practice and experiment, and not upon a knowledge of principles. No one who is ignorant of his own language, which is the means of conveying ideas, can profitably investigate or study science. In the healing art, the most thorough study and investigation of very profound subjects are necessary.

Again, any professional man must possess a good education, to command respect and influence in society. The ignorant pretender always deserves, and usually receives the scorn and contempt of the enlightened public.

The suggestions in regard to the classification of studies is a matter of the utmost importance, especially when considered in reference to a course of study in a Dental college.

All who have been engaged in teaching know full well that it is wholly impossible to study with profit nine or ten distinct subjects at the same time, and yet such is the attempt in the ordinary course of instruction in our Dental colleges.

The proper classification and arrangement of the different branches of the course, has been one of the greatest defects in our system of education, both in the private office and in the schools; and though the classification suggested here is not just what we think it should be in the Colleges, still it is a great step in the right direction; and perhaps is about right for private study. Four or five distinct subjects are as many as *any* one can manage at once. The stuffing method heretofore pursued, does injustice both to the student and to the profession, and should at once be abandoned, and a more rational course be adopted.

It affords us much gratification to state that these suggestions are being very numerously endorsed by the profession.

---

The American Dental Association, at a meeting held in Boston, beginning July 31st, 1866, passed the following Resolution:

"*Resolved*, 1st. That a Committee of three be appointed to draft suitable suggestions upon the subject of accepting students, and that such suggestions be printed in circular form for the consideration of every Dental practitioner in the United States.

"2d. That the expense of such printing and distribution be borne by the Association."

The undersigned, the Committee appointed according to the provisions of the above Resolution, are fully persuaded that the time has now arrived when every Dental practitioner in the country can and should lend his aid in elevating the status of the profession, to the end that those who are soon to fill our places, may be prepared in a greater degree, to fulfill the reasonable expectations of the public and hold Dentistry in its proper rank among the learned professions.

Our Dental colleges have done much, and will doubtless do more, but there is a work for the private instructor to accomplish, that students may be better qualified to enter such collegiate institutions and graduate with credit to themselves and honor to the profession. It is not only essential that Dental colleges exist, but they should be furnished with properly qualified pupils to ensure that success and usefulness contemplated in their foundation.

Relying, then, upon the generous co-operation of our professional brethren, we respectfully submit the following "suggestions" as a basis in "accepting Dental students:"—

1st. He must possess a good moral character and at least a good English education.

2d. He must be required to apply himself diligently for three years, including two full courses of lectures in some Dental college, to the following studies, viz.:—

*First Year*—ANATOMY, HISTOLOGY AND PHYSIOLOGY.

*Second Year*—PATHOLOGY, CHEMISTRY, METALLURGY AND MECHANICAL DENTISTRY.

*Third Year*—OPERATIVE DENTISTRY, SPECIAL PATHOLOGY, DENTAL MEDICINE AND MICROSCOPY.

We further suggest that the instructor examine his pupil in his studies at least twice in every week, and as much oftener as may be convenient,—not, however, including his lecture terms; and should the student, after sufficient trial, fail to exhibit the necessary talent for our specialty, he should be kindly apprised of the fact, and advised to seek other fields of usefulness.

---

Practitioners favoring the foregoing are respectfully requested to date and sign the accompanying paper, and forward to the Committee.

A. LAWRENCE, LOWELL, MASS.
C. P. FITCH, NEW YORK CITY.
J. TAFT, CINCINNATI, OHIO.

*January 10th*, 1867.

